# Autologous chondrocyte implantation in osteochondral defects of the capitulum: clinical and radiological outcome in a case series

**DOI:** 10.1016/j.jseint.2026.101770

**Published:** 2026-07-26

**Authors:** Anton F. Schmidt, Isabella Weiß, Doruk Akgun, Marie Reisener, Lucca Lacheta, Marvin Minkus, Alexander Spöck, Stephan Pauly, Kathi Thiele

**Affiliations:** a Medical University of Vienna, Vienna, Austria; b Clinic for Shoulder- and Elbow surgery, Auguste Viktoria Klinikum Vivantes, Berlin, Germany; c Center for Musculoskeletal Surgery, Berlin, Germany; d Orthopassion, Privat Practice for Orthopedics, Freiburg, Germany; e Department of Sports Orthopaedics, Technical University of Munich, Klinikum Rechts der Isar, Munich, Germany; f Clinic for Diagnostic and Interventional Radiology, Auguste Viktoria Klinikum Vivantes, Berlin, Germany

**Keywords:** Autologous chondrocyte implantation, ACI, Cartilage defect, Osteochondral lesion, Capitulum, Elbow, Chondrocyte spheroids, Cartilage repair

## Abstract

**Background:**

Osteochondritis dissecans (OCD) of the capitellum is a cartilage and subchondral bone disorder predominantly affecting young, physically active individuals. While established surgical techniques such as microfracturing and osteochondral autograft transfer offer acceptable outcomes, limitations in cartilage quality or procedural invasiveness remain. Autologous chondrocyte implantation (ACI) has been proposed as a less invasive, biologically restorative alternative, though clinical data on its use in the elbow are not available. Therefore, the aim of this pilot study was to evaluate the feasibility of ACI for the treatment of elbow OCD in a case series.

**Methods:**

In this prospective observational study, 6 male patients (20.5 ± 5.2 years) with centralized International Cartilage Research Society grade IV OCD lesions of the capitellum were included and treated with ACI. Clinical evaluations were performed at 6 months (T1), 12 months (T2), and 34.8 ± 7.9 months (T3) post-operatively, including assessment of range of motion, subjective elbow value, visual analog scale for pain, Mayo Elbow Performance Score, Kerlan-Jobe Orthopaedic Clinic score, and return to sport. Magnetic resonance imaging was conducted at T2 and T3, and cartilage repair tissue was analyzed using the magnetic resonance observation of cartilage repair tissue 2.0 Ankle Score.

**Results:**

Improvements were observed across all clinical outcome measures. Subjective elbow value increased from 59 ± 18.5% pre-operatively to 91.2 ± 4.2% at final follow-up, while visual analog scale under load decreased from 6.5 ± 1.6 to 0.8 ± 0.7. Mayo Elbow Performance Score and Kerlan-Jobe Orthopaedic Clinic improved from 70.6 ± 17.7 to 97.5 ± 6 and from 38.3 ± 10 to 88.5 ± 8.4, respectively. Magnetic resonance imaging demonstrated defect filling and integration in all individuals, as assessed by the magnetic resonance observation of cartilage repair tissue score, which showed no deterioration between T2 and T3. The mean return to sport time was 6.8 ± 3 months. No complications or need for revision surgery were recorded.

**Conclusion:**

This pilot feasibility study provides preliminary insights into the treatment of centralized capitellar OCD lesion with ACI in a case series of young, active patients, with clinical and radiological findings at a mean 3-year follow-up (range, 25–42 months). These exploratory findings suggest that ACI may warrant further investigation for focal, symptomatic elbow defects with minimal or limited involvement of the subchondral bone. Larger controlled studies are needed to define the role of ACI relative to established treatment options.

Osteochondritis dissecans (OCD) of the capitellum is a condition primarily affecting adolescent and young adult athletes, particularly those engaged in repetitive overhead or axial-loading sports, with a prevalence of 1-3% in young baseball players.^17^^,^^19^ The disease affects both articular cartilage and subchondral bone, and the primary therapeutic goals are the restoration of joint function, prevention of secondary osteoarthritis, and a safe return to sport (RTS).

Although evidence for a standardized therapeutic algorithm is lacking, conventional surgical options include arthroscopic débridement, microfracturing, fragment fixation, and osteochondral autograft transfer (OAT).^10^ Lesion characteristics such as size, stability, containment, and location influence the choice and success of each surgical approach.^3^^,^^19^^,^^20^^,^^28^^,^^32^ Microfracturing aims to stimulate fibrocartilage formation through bone marrow stimulation but fails to address the subchondral architecture.^1^^,^^31^ This limitation likely contributes to inferior outcomes in high-demand athletes in terms of RTS.^3^^,^^6^^,^^12^^,^^15^^,^^16^^,^^31^ Westermann et al^31^ reported RTS to preinjury levels in only 71% of patients following microfracturing, compared to 94% with restorative procedures such as OAT. OAT offers the advantage of reconstructing both the cartilage and the underlying subchondral bone, which may explain its higher success rates, especially in larger and lateralized defects.^14^^,^^16^^,^^17^^,^^24^^,^^28^^,^^29^ However, it remains a relatively invasive procedure with potential donor site morbidity.^2^

In this context, autologous chondrocyte implantation (ACI) could present an alternative for central defects with low subchondral involvement. Although well established in larger joints such as the knee, its use in the elbow remains limited, and there are only technical notes for the elbow.^11^^,^^22^^,^^23^ ACI enables biological regeneration of hyaline-like cartilage and may be particularly suitable for young, high-demand patients with centralized lesions and minimal subchondral bone loss. When necessary, concomitant subchondral bone augmentation with lesions exceeding 3 mm, as routinely performed in the knee, could potentially enhance outcomes. Given the biomechanical demands placed on the radiocapitellar joint, identifying optimal indications and limitations for ACI in this population is of considerable clinical relevance.^31^ The aim of this pilot feasibility case series was to descriptively evaluate clinical, functional, and radiological outcomes after ACI for capitellar OCD lesions and to report individual patient trajectories over midterm follow-up.

## Materials and methods

This prospective observational pilot feasibility study included patients treated between 2019 and 2022 at an academic single center specializing in elbow surgery. Eligible chondral or osteochondral lesions were diagnosed pre-operatively using radiographs and magnetic resonance imaging (MRI).

Inclusion criteria were defined as isolated acute or chronic focal cartilage defects (traumatic or related to OCD), International Cartilage Research Society Grade IV, centralized, contained defects, closed epiphyseal state on the ipsilateral distal humerus, and low subchondral defect. Inclusion was only performed if the patient characteristics appeared suitable for ACI, taking into account patient age, lesion size, and depth.

Exclusion criteria included kissing lesions of the radial head, cubital osteoarthritis (Kellgren-Lawrence > II), grade IV lesions with large osseous defects (depth >3 mm), posttraumatic malformations, and lateralized, non-contained defects.

Evaluation was conducted pre-operatively (T0), 6 months (T1), 12 months (T2), and at final follow-up (T3, min. 25 months) post-operatively. Evaluation included a subset of tools; for further information, see Table I.

**Table I:** Measurements at follow-up time points.

Assessment	T0 pre-operatively	T1 6 mo	T2 1 yr	T3 final
Range of motion (ROM)	✓	✓	✓	✓
Specific tests∗	✓	✓	✓	✓
SEV (subjective elbow value)	✓	✓	✓	✓
VAS (rest and activity)	✓	✓	✓	✓
MEPS (Mayo Elbow Performance Score)	✓	✓	✓	✓
KJOC (Kerlan-Jobe Orthopaedic Clinic Score)	✓	✓	✓	✓
Return to sport evaluation†		✓	✓	✓
Radiographic assessment (MRI)	✓		✓	✓

*VAS*, visual analog scale; *MRI*, magnetic resonance imaging.

∗ Moving valgus test, pincer grip test, osteochondral shear test.

† Return to play, time to return to play, return to preinjury level of play or higher, return to active participation without specifics on level, inability to return to play, return to play with lower participation level, return to play limitations including performance decline or pain.

The primary composite clinical endpoint was defined as the proportion of patients with unrestricted elbow range of motion and pain visual analog scale (VAS) 0-1 under load at final follow-up. This endpoint was selected to describe a pragmatic state of near pain-free functional recovery, combining 2 clinically relevant domains after capitellar OCD treatment in young and active patients: unrestricted elbow motion and absence of relevant load-related pain. A VAS threshold of 0-1 was chosen to reflect no or only minimal pain under load. As this composite endpoint has not been validated specifically for elbow cartilage restoration, it was interpreted descriptively and in conjunction with established clinical and patient-reported outcome measures, including Mayo Elbow Performance Score, Kerlan-Jobe Orthopaedic Clinic (KJOC), subjective elbow value (SEV), VAS pain, and RTS.

Secondary endpoint 1: The proportion of patients who, on post-operative MRI at T2 and T3, demonstrate load-stable cartilage repair tissue meeting all of the following criteria: thickness and surface comparable to adjacent healthy cartilage, complete integration into the surrounding chondral structure, absence of adhesions, and intact subchondral bone plate with normal underlying bone marrow.

Secondary endpoint 2: The proportion of patients who return to their pre-operative sports performance level. In this context, “return to play” and “time to return to play” are assessed and compared.

### Operative technique

Depending on concomitant injuries and the preferred cartilage harvest method, an index arthroscopy for cartilage harvesting was performed either at the ipsilateral elbow joint (eg, extraction of a cartilage flake and/or management of intra-articular pathology) or at the knee joint. For elbow arthroscopy, patients were positioned in the prone position, and standard arthroscopic portals were established, including the anteromedial, anterolateral, proximal posterolateral, distal posterolateral, and direct posterior portal. Index arthroscopy also enabled intraoperative assessment of the subchondral bone condition and classification of the cartilage defect according to the International Cartilage Research Society classification.

Depending on the underlying pathology, the following options for harvesting potential cartilage cells were chosen:

1. For traumatic origin and OCD: Cartilage cells can be harvested from free, non-reimplantable/non-refixable cartilage fragments or from the dissecate^8^^,^^9^ (Fig. 1*B*).

**Figure 1:**
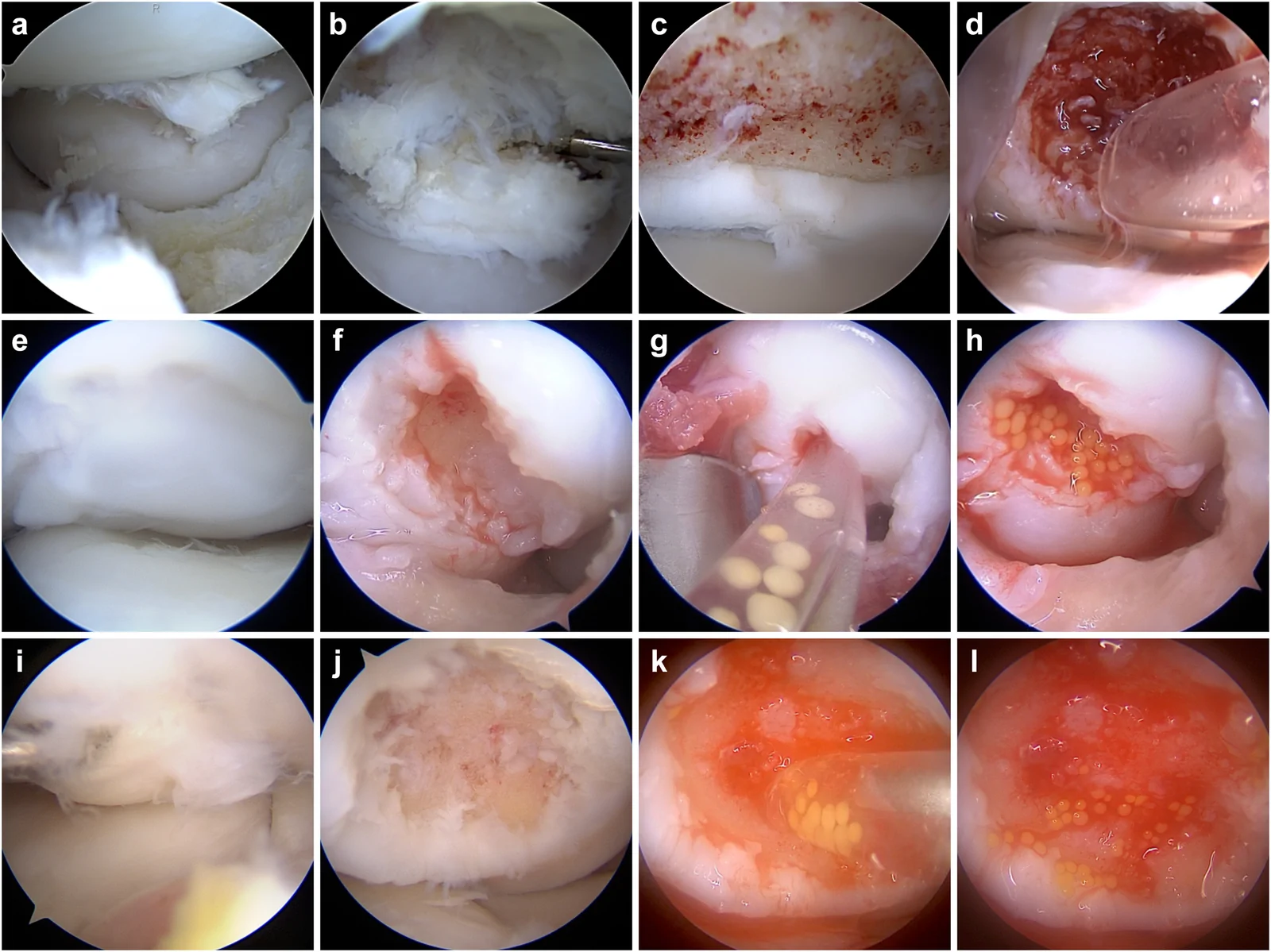
Arthroscopic autologous chondrocyte implantation. (**a, e, i**) Assessment of defect size. (**b, c, f, j**) Defect appearance during and after débridement and preparation. (**d, g, h, k, l**) Implantation of 3-dimensional spheroids into the defect.
2. From minor weight-bearing areas of the knee (notch of the lateral femur condyle): This area provides an alternative source of cartilage cells while minimizing the impact on joint function.
3. From the radial head: In exceptional cases requiring an open approach to address concomitant injuries, harvesting from the radial head may be performed.

The small sample of chondrocytes was then cultivated and processed (co.don AG ACI Product Germany) for 6 to 8 weeks. The final product consists of autologous cells encapsulated in an endogenously produced extracellular matrix forming spherical aggregates (chondrocyte spheroids). The spheroids were then implanted arthroscopically via the posterolateral portal (Fig. 1) or mini-open technique (anconeus splitting approach) to cover the cartilage defect. Implantation was performed under dry conditions after complete evacuation of irrigation fluid to ensure stable adherence of the spheroids, which were carefully applied to the prepared defect bed using a cannula provided by the manufacturer.

### Clinical, functional, and radiological evaluation

Post-operative MRI was used to evaluate the cartilage repair tissue at follow-up T2 and T3. MRI acquisition protocols varied between patients and scanners (Siemens, 3T, pd_tse_fs, pd_tse_spair, 3 mm slice thickness; Optima, 1.5 T, fw_pd_fs, 1 mm slice thickness), which limits the comparability and precision of MRI-based measurements. In addition to a binary assessment of defect filling, the magnetic resonance observation of cartilage repair tissue (MOCART) 2.0 Ankle Score was applied as an exploratory and descriptive tool to provide a more structured evaluation of the cartilage repair tissue. The ankle-specific version was selected over the knee-specific MOCART 2.0 score because it places greater emphasis on osteochondral defect morphology and subchondral bone-related parameters, including defect volume fill, bony defect or overgrowth, edema-like marrow signal, and subchondral cysts. These features were considered relevant for the assessment of capitellar OCD lesions. This scoring system allows for an analysis of cartilage repair tissue, including parameters such as defect fill, integration to the border zone, surface structure, signal intensity, and subchondral bone changes. Although the MOCART 2.0 Ankle Score has not been validated for use in the elbow joint, it was applied in this study at follow-up time points T2 and T3 only to enable structured intraindividual comparison of MRI findings over time. No direct comparison of MOCART results between individual patients was performed. Due to the non-validated use of this score in the elbow and the heterogeneity of MRI scanners, slice thickness, and acquisition protocols, the radiological findings should be interpreted with caution. This approach was intended to provide a standardized descriptive assessment of repair tissue morphology over time rather than evidence of long-term efficacy or durability of the cartilage repair technique.

## Results

### Patients

A total of 6 male patients (mean age 20.5 ± 5.2 years) with grade IV centralized OCD lesions of the capitellum were included (Table II). The follow-up included the time points 6 months (T1), 12 months (T2), and 34.8 ± 7.9 months (T3; min. 25 months, max. 42 months) post-operatively. Cartilage harvest for ACI was performed from the ipsilateral knee joint (n = 4, ID: 2,3,5,6), radial head (n = 1, ID: 4), and directly from the osteochondral fragment (n = 1, ID: 1). The ipsilateral knee was used as the standard harvest site in 4 patients, while the radial head was selected in one patient during open reduction and screw fixation of the osteochondral fragment to avoid additional donor-site morbidity; in another patient, the osteochondral fragment was sufficiently large for cell harvest but not suitable for refixation. Two out of 6 patients reported a recollectable traumatic event prior to symptom onset. One of these patients (ID: 1) presented with a concomitant bony avulsion fracture of the lateral collateral ligament, which required no further surgical intervention, while another patient (ID: 4) underwent open reduction and internal fixation of an osteochondral fragment using a headless compression screw (DePuy Synthes), with a persistent central defect already evident during the initial procedure. Therefore, the decision was made to harvest cells during the first surgery and cover the defect in a second surgery. The mean defect size was 8.3 ± 2.0 mm × 7.3 ± 2.7 mm (ø 0.63 cm^2^), with no need for subchondral bone grafting as all lesions fulfilled the predefined inclusion criterion of ≤3 mm subchondral involvement. All lesions were considered contained and stable following initial débridement.

**Table II:** Patient, lesion, and return-to-sport characteristics.

ID	Age∗	Sex	BMI	Dominant/injured side	Concomitant injury	Defect size (cm)	Cartilage harvest	Sports	Level of sport	RTS (mo)
1	30	m	28.6	r/r	Bony LCL lesion	1.2 × 0.8	Free chondral flake	Tennis	Hobby	7
2	22	m	22.6	r/l	None	0.8 × 0.8	Knee	Climbing	Hobby	6
3	19	m	19.8	r/r	None	0.6 × 0.2	Knee	Fitness	Hobby	3
4	17	m	20.7	r/r	ORIF osteochondral fragment†	0.8 × 0.8	Radial neck	Handball	Competitive	5.6
5	20	m	23.0	r/r	None	0.8 × 1.0	Knee	Soccer	Competitive	7
6	15	m	28.6	r/r	None	0.8 × 0.8	Knee	Handball	Competitive	12

*ID*, patient identification; *m*, male; *r*, right-handed; *l*, left-handed; *LCL*, lateral collateral ligament; *RTS (mo)*, return to sport in months; *ORIF*, open reduction and internal fixation; *BMI*, body mass index.

∗ Age at surgery in years.

† Fixation in the index surgery.

### Clinical outcomes

Improvements were observed across the cohort in the majority of clinical outcome measures, including increased range of motion, higher SEV scores, and decreased pain under load (Table III). At final follow-up, 4 of 6 patients reached the predefined primary clinical endpoint of VAS ≤1 under load and unrestricted range of motion. This outcome remained consistent throughout the follow-up period from T2 to T3 in these patients (Table III). Among the 6 patients, 2 patients demonstrated positive disease-specific tests pre-operatively (osteochondral shear test; ID: 1,2). At T3, only one patient showed a minimal residual finding in the pincer-grip test (ID: 1), whereas all other patients showed no positive disease-specific testing. Pre-operatively, 3 patients demonstrated an extension deficit of 20 ± 10° (ID: 1,3,6). While this improved in 2 of them (ID: 1,6), one patient (ID: 3) with an initial 10° deficit showed a progression to a 30° extension deficit at both the T2 and T3 follow-up. This patient also reported sport-specific limitations; however, patient-reported scores remained stable between T2 and T3 (SEV: 98% and 90%; Mayo Elbow Performance Score: 100 and 100; KJOC: 92.8 and 81). No post-operative complications (infection, graft delamination, revision surgery) were observed during follow-up. No patient developed clinically relevant post-operative stiffness requiring additional treatment. Among the 4 patients in whom cartilage was harvested from the ipsilateral knee joint, no donor-site morbidity or knee-related complaints were reported at final follow-up (T3).

**Table III:** Individual clinical outcome scores.

ID	SEV (%)				KJOC				MEPS				VAS at rest/under load			
	T0	T1	T2	T3	T0	T1	T2	T3	T0	T1	T2	T3	T0	T1	T2	T3
1	30	90	92	90	25.5	96.9	67	77	44	100	100	85	0/8	1/2	0/1	0/1
2	60	75	100	98	24.3	46.3	68	88.5	85	100	100	100	1/8	0/7	0/2	0/1
3	60	95	98	90	40	60	92.8	81	85	100	100	100	1/4	0/1	0/1	0/1
4	70	93	89	93	39.9	65.8	90.5	99	70	90	100	100	2/7	0/2	0/1	0/0
5	50	80	81	85	52.5	44.9	66	96	55	85	100	100	0/7	0/1	0/1	0/2
6	85	80	95	91	36.6	40	32.1	89.5	85	100	100	100	0/5	0/2	0/1	0/0
Mean	59	86	93	91	38.6	59	69	88.5	70.6	96	100	97.5	0.6/6.5	0.17/2.5	0/1	0/0.83

*SEV*, subjective elbow value; *VAS*, visual analog scale; *MEPS*, Mayo Elbow Performance Score; *KJOC*, Kerlan-Jobe Orthopaedic Clinic Score.

Given the small number of patients, these findings should be interpreted descriptively at the individual patient level rather than as evidence of statistically robust treatment effects.

### Return to sports

The mean time to RTS was reported as 6.8 ± 3 months post-operatively in the overall cohort of 6 patients (Table II). Competitive athletes required a longer period, with a mean RTS of 8.3 months. However, 3 of 3 competitive athletes (2 overhead athletes, 1 football player) were able to regain their pre-operative or higher performance level at the 1-year follow-up. None of these 3 patients reported elbow-related issues during sport-specific activity. At the 3-year follow-up, 1 of 3 competitive athletes (ID: 5) was playing in a lower division than pre-operatively; however, this was not attributed to elbow-related limitations. In contrast, recreational athletes (tennis, climbing, fitness) returned to sport earlier, with a mean RTS of 5.3 months. At both the 1- and 3-year follow-ups, 2 of 3 recreational athletes (ID: 2,3) reported not having reached their pre-operative performance level, which they attributed to non-elbow-related factors, such as time constraints. One of these patients (ID: 3), who presented with a persistent 30° extension deficit, reported elbow-related discomfort during sporting activity. Regarding workability, all 6 patients rated their work ability as good 1 year post-operatively. However, 1 of 6 patients (ID: 2) reported residual elbow pain during high-load activities such as push-ups. The validated German version of the KJOC score,^26^ which includes a threshold value for asymptomatic overhead athletes, was used in this study and showed that at T3, all 6 patients exceeded the threshold of 68.6, with a mean score of 88.5 ± 8.4, indicating KJOC values above the proposed threshold for asymptomatic overhead athletes.^21^

### Radiographic outcomes

**Figure 2:**
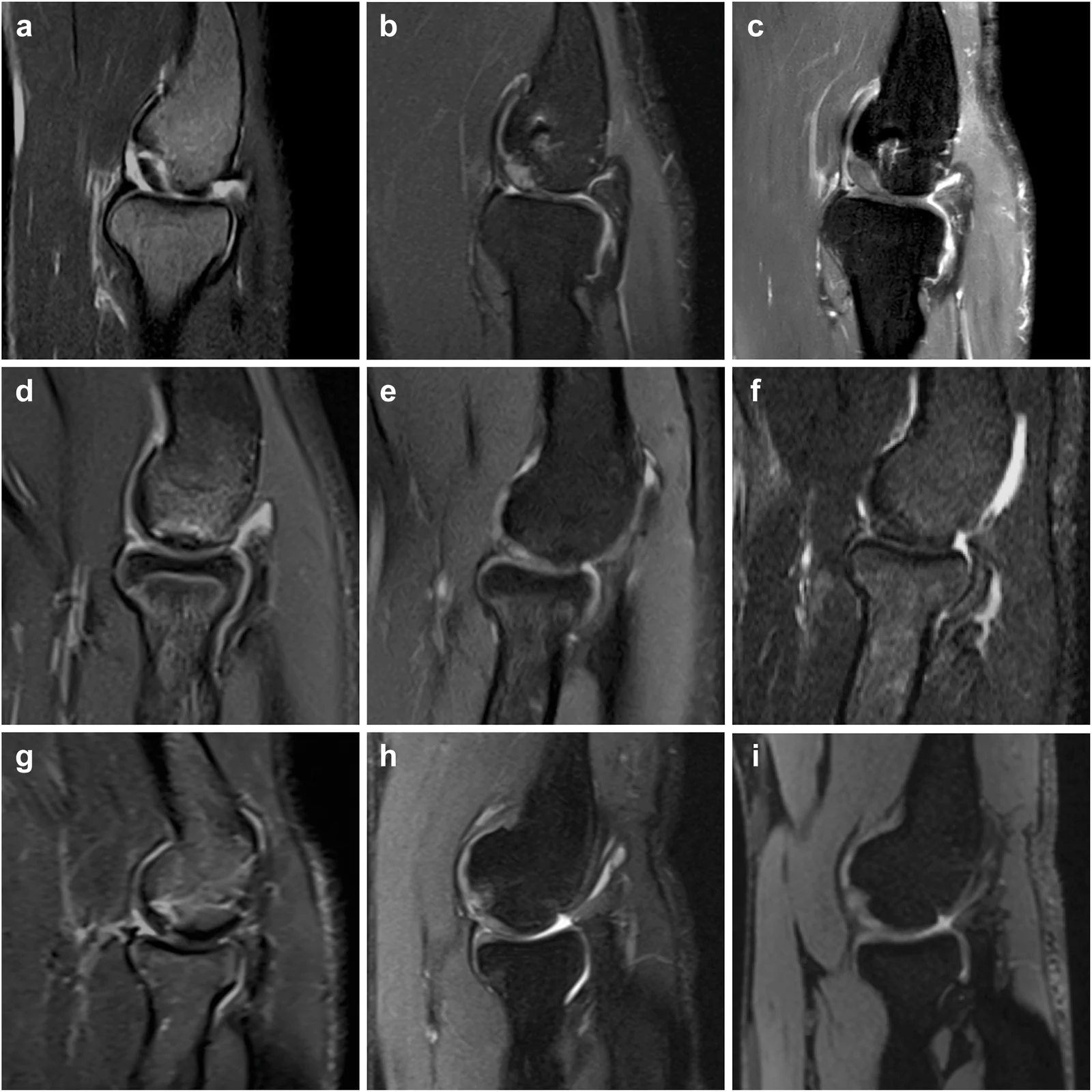
Sagittal MRI sections of 3 representative patients, each displayed in a single row, demonstrating the cartilage defect of the humeral capitellum pre-operatively (**a, d, g**) and the repair tissue at T2 (**b, e, h**) and T3 (**c, f, i**). *MRI*, magnetic resonance imaging.

**Figure 3:**
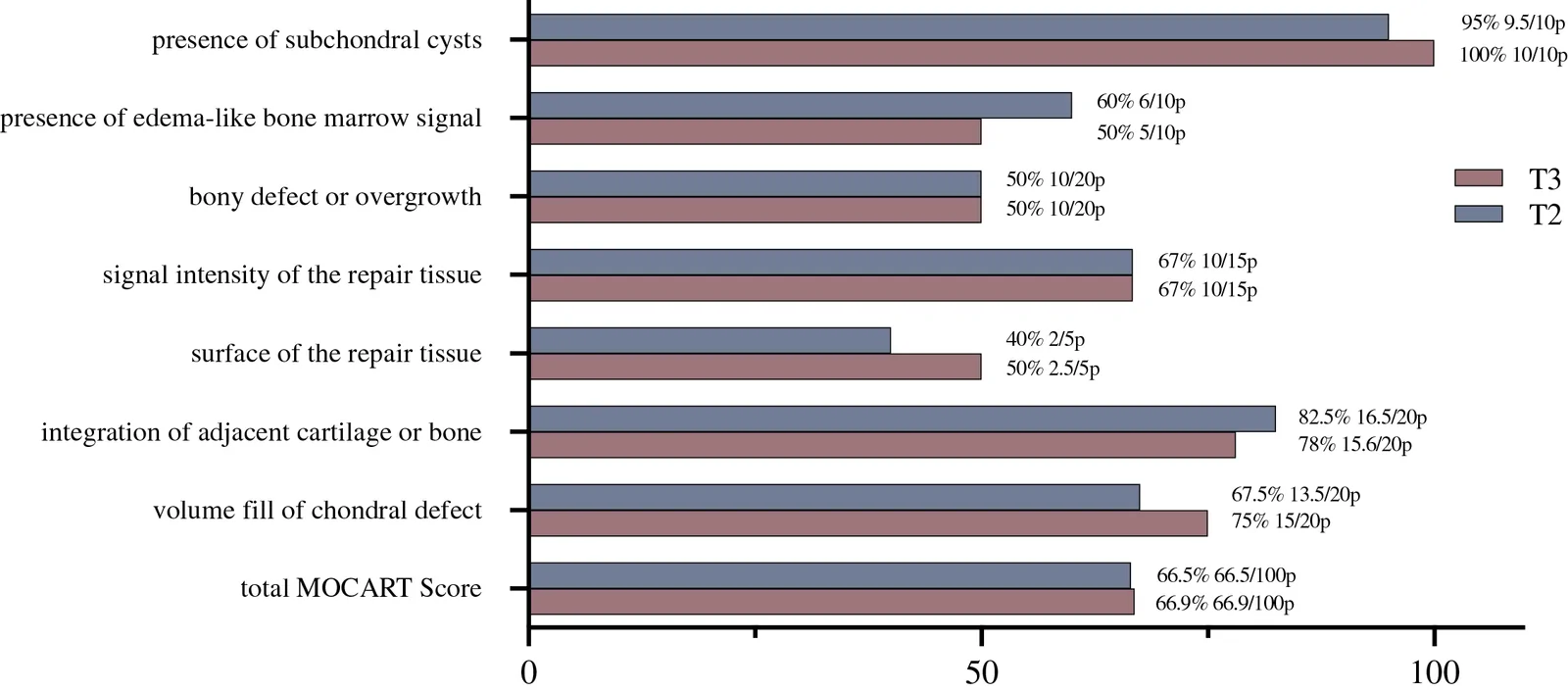
Magnetic resonance observation of cartilage repair tissue score at T2 and T3.

Pre-operative MRI revealed defects measuring 8.3 × 7.3 mm (ø 0.63 cm^2^) on average (Fig. 2*A, D*, and *G*). Evaluation at both 1- and 3-year follow-ups demonstrated MRI-based signs suggestive of cartilage defect filling, insofar as image quality allowed (Fig. 2, *B, C, E, F, H* and *I*). Two patients (ID: 4,5) demonstrated mild cartilage hypertrophy on imaging (Fig. 2, *B* and *C*). However, these 2 patients did not show inferior outcomes in clinical scores, and no clinical correlation with the observed cartilage hypertrophy could be established. One patient (ID: 4, Fig. 2, *A*–*C*) received screw fixation of an osteochondral fragment. The MOCART 2.0 Ankle Score was used as an exploratory, non-validated tool for structured MRI assessment in the elbow and was assessed separately by 1 senior radiologist and 1 senior orthopedic surgeon, showing moderate interrater reliability with an intraclass correlation coefficient of 0.67 (intraclass correlation coefficient, 2-way random effect, 95% confidence interval). The MOCART evaluation (Fig. 3) showed defect filling of the osteochondral defect (T2: 13.5/20; T3: 15/20) and integration into adjacent cartilage or bone at both time points (T2: 16.5/20; T3: 15.6/20), suggesting incorporation of the repair tissue on MRI. In contrast, the surface integrity (T2: 2/5; T3: 2.5/5) and signal intensity (T2 and T3: 10/15) showed comparatively lower scores, which may indicate irregular repair morphology and incomplete tissue maturation. The presence of a bony defect or overgrowth contributed to reduced subscores in this category (T2 and T3: 10/20), possibly related to graft hypertrophy. Within the limitations of heterogeneous MRI protocols and the non-validated use of the MOCART 2.0 Ankle Score in the elbow, no decline was observed in the overall MOCART scores between T2 and T3 (66.5/100 vs. 66.9/100), suggesting no apparent deterioration of the MRI-based repair tissue appearance over this period.

## Discussion

This study provides preliminary clinical data and, to our knowledge, represents the first report on the feasibility of ACI for capitellar osteochondral defects of the elbow. In this small pilot feasibility case series, generally favorable clinical and radiological findings were observed during follow-up, and no revision surgery was required. However, given the very small cohort size and uncontrolled study design, these observations should be interpreted as exploratory and hypothesis-generating rather than evidence of treatment efficacy. The findings may support further investigation of ACI in selected young and active patients with focal capitellar defects and minimal or limited subchondral bony involvement.

Key questions and findings remain, particularly regarding the comparative effectiveness of treatment options. The therapeutic approaches may be better understood as complementary to one another rather than competing, each offering specific advantages depending on the indication. While ACI is technically demanding, cost-intensive and performed as a 2-stage procedure, it offers the potential to restore hyaline-like cartilage, which may be especially beneficial in patients with high functional demands on the elbow. Compared to microfracturing, ACI is capable of preserving joint architecture better and offers a less invasive alternative to OAT in selected cases of the knee.^4^^,^^13^^,^^22^ Evidence from other joints indicates that defect size is a decisive factor in determining the indication for regenerative cartilage repair.

In the elbow joint, additional factors such as athletic activity level and mechanical joint demand may play an equally important role in determining the indication for cartilage repair. Consequently, young and physically active patients with appropriately sized focal defects may represent a subgroup in whom ACI warrants further investigation, particularly when biological cartilage repair and preservation of joint architecture are considered relevant treatment goals. Prospective studies are warranted to define the optimal treatment algorithm and clarify the potential role of regenerative techniques, such as ACI and minced cartilage, across different patient cohorts. At present, the implantation of osteochondral grafts harvested from the knee joint or the ribs is an established treatment option, although the invasiveness of addressing a different joint and the proportional lack of compatibility of cartilage thicknesses are subjects of debate.^2^^,^^18^^,^^25^^,^^27^^,^^30^ There is currently no optimal solution, particularly for laterally located defects, as outcomes may depend on lesion containment, defect size, subchondral bone involvement, and the presence of a stable cartilage rim. Whether ACI may represent an alternative solution cannot be clarified with this study design, as only central, contained defects were considered.

In the present cohort, the mean time to RTS was 6.8 ± 3 months. Although previously published studies on microfracturing and OAT have reported varying RTS timelines and rates, direct comparisons with the present findings are not possible due to differences in patient selection.^3^^,^^5^, ^6^, ^7^^,^^12^^,^^14^, ^15^, ^16^^,^^31^ In this case series, all competitive athletes (n = 3) were able to return to their previous or an even higher level of athletic performance. It should also be noted that 2 of 3 recreational athletes did not return to their previous level of sport. Although both reported reasons unrelated to their elbow condition, a potential contribution of residual joint limitations cannot be completely ruled out.

This study has several limitations that must be acknowledged. As a case series with a very small sample size and midterm follow-up, the generalizability of the findings is limited, and no meaningful inferential statistical analysis can be performed. In addition, substantial selection bias must be considered, as only highly selected patients with centralized, contained defects, minimal or limited subchondral involvement, and young, active patient profiles were included. This represents a favorable subgroup for cartilage repair and limits the external validity of the findings. Therefore, the results cannot be generalized to patients with lateral or uncontained lesions, larger osteochondral defects, relevant subchondral bone loss, lower activity levels, or older age. Furthermore, interpretation of the MRI results and the MOCART 2.0 Ankle Score should be viewed with caution due to heterogeneous imaging protocols, different MRI scanners, and variable slice thicknesses ranging from 1 to 3 mm. Although the MOCART 2.0 Ankle Score has not been validated for the elbow joint, it was used exploratively to allow structured intraindividual comparison of repair tissue morphology between T2 and T3; no direct comparison between patients was intended. Finally, the resilience and long-term durability of the newly generated cartilage after ACI cannot be definitively assessed after medium-term follow-up. Only long-term data can determine whether ACI, which involves significantly higher costs and is a 2-stage procedure, may offer advantages in selected lesions compared with other techniques.

## Conclusion

In this pilot feasibility case series, ACI using 3-dimensional spheroids of autologous matrix-associated chondrocytes for osteochondral defects of the capitellum was associated with stable clinical and radiological findings at a mean 3-year follow-up (range, 25-42 months) for all 6 patients. These preliminary findings suggest that ACI may represent a feasible approach for a highly selected patient cohort with focal, symptomatic elbow defects and minimal or limited subchondral involvement. However, given the very small cohort size and uncontrolled study design, these results should be interpreted as exploratory. Future comparative studies are needed to further clarify the potential role of ACI among available cartilage repair techniques.

## Disclaimers:

Funding: Because the costs of ACI at the elbow are not covered by German health insurers, Co.don GmbH (Teltow, Germany) covered the treatment costs. Co.don GmbH had no role in study design, data collection, data analysis, decision to publish, or preparation of the manuscript.

Conflicts of interest: The authors, their immediate families, and any research foundations with which they are affiliated have not received any financial payments or other benefits from any commercial entity related to the subject of this article.
